# Implementation outcomes of 1-3-7 focus investigation for malaria in a low transmission setting in Southern Province, Zambia: A mixed methods study

**DOI:** 10.1371/journal.pgph.0004179

**Published:** 2025-01-17

**Authors:** Anne C. Martin, Japhet Matoba, Caison Sing’anga, Mukuma Lubinda, Sydney Mweetwa, Xinyue Chen, Ignatius Banda, Busiku Hamainza, Harry Hamapumbu, Edgar Simulundu, William J. Moss, for the Southern and Central Africa International Centers of Excellence for Malaria Research

**Affiliations:** 1 Department of Epidemiology, Johns Hopkins Bloomberg School of Public Health, Baltimore, Maryland, United States of America; 2 Macha Research Trust, Choma, Zambia; 3 Department of International Health, Johns Hopkins Bloomberg School of Public Health, Baltimore, Maryland, United States of America; 4 National Malaria Elimination Centre, Zambia Ministry of Health, Lusaka, Zambia; 5 Department of Molecular Microbiology and Immunology, Johns Hopkins Bloomberg School of Public Health, Baltimore, Maryland, United States of America; Monash University Indonesia, INDONESIA

## Abstract

Eleven countries have been certified as malaria free since 2016, but none of these are in subSaharan Africa where elimination challenges are unique. The 1-3-7 focus investigation approach is an implementation strategy that requires case reporting, case investigation/classification, and focal classification/response to be completed one, three, and seven days, respectively, after index case diagnosis. Real-time short-messaging-service reports are sent at each step to add accountability and data transparency. Reactive case detection is one focal response of the 1-3-7 strategy. China, Thailand, Myanmar, and other countries cite high fidelity to deadlines and broad acceptability of 1-3-7, but this strategy has yet to be widely deployed in Africa. This mixed-methods study evaluated implementation and service outcomes of 1-3-7 focus investigation in a rural area of southern Zambia. Selected outcomes were fidelity, efficiency, feasibility, equity, and acceptability, assessed via program metadata and semi-structured interviews with program personnel. Fidelity was moderate with 61% of cases reported. Focus investigation and reactive case detection completion doubled in areas using 1-3-7, from 20% to 42%. However, reactive case detection, which involved screening community members residing within 140 meter of index cases with a rapid diagnostic test, detected few parasitemic individuals, suggesting this may not be the most efficient day 7 response in this setting. Mobile phone network coverage was a common challenge to feasibility that likely affected reporting rates and fidelity. Thirty-four percent of health-facility diagnosed cases were not eligible for 1-3-7 follow-up. Distance from the health center was a barrier to feasibility and equitable reach of services. Reporting was faster in areas where health workers classified transmission as higher and slower in areas with poor mobile phone network coverage. The strategy was widely accepted. Scale-up should include adherence-focused management strategies, spatially targeted interventions not reliant on RDTs, and complementary surveillance that targets hard-to-reach populations.

## Background

The World Health Organization (WHO) Global Technical Strategy for Malaria 2016–2030 incorporates ambitious global targets including malaria elimination in 35 additional countries by 2030 [1]. Since 2016, eleven countries (Algeria, Argentina, Azerbaijan, Belize, Cabo Verde, China, El Salvador, Kyrgyzstan, Paraguay, Tajikistan, Uzbekistan), have been WHO-certified as malaria free. Each has a strong primary health care system that: 1) provides access to diagnosis and treatment; 2) uses targeted vector control in response to local transmission; 3) has a dedicated community-based workforce; and 4) conducts decentralized surveillance for early case detection [2–9]. Surveillance is a pillar of the WHO’s technical approach and 1-3-7 focus investigation is an implementation strategy recommended by the WHO that uses surveillance tools and deadlines to provide a framework for delivering evidence-based interventions in low transmission settings [1]. While widely utilized in South-East Asia, this strategy has seen little use on the African continent, even in parts of countries where successful malaria control campaigns have reduced incidence to levels that make these areas eligible for elimination-focused malaria interventions [10–15]. WHO recommends countries tailor interventions sub-nationally so that provinces or districts within the country receive strategies that are effective given their malaria burden; thus, elimination status is often tracked by countries at district or provincial levels [15].

While often described as an intervention, 1-3-7 focus investigation is a strategy for delivering case-based reactive interventions that have long been recommended in low-transmission areas. The strategy is simply the installation of a reporting and surveillance platform that standardizes response times for index case notification, index case classification, and service delivery of evidence-based interventions (i.e., focus area investigation and response) [1]. Notification of an incident case of malaria in a focus area is sent within one day of diagnosis (day 1). By day 3, case classification occurs using schema that identify cases as indigenous or imported (China, Cambodia), or local indigenous, introduced indigenous, intraported, or imported (Myanmar, Thailand, Vietnam) [10–14]. By day 7, the transmission intensity of the focus area is classified using malaria incidence, recency (within the past year, past three years, or three or more years) of the last locally transmitted case, and the presence of malaria vectors [10–14]. By day 7, a responsive evidence-based intervention should be conducted based on the case and focus classification and what interventions are available in country. Interventions include focal indoor residual spraying, distribution of long-lasting insecticide treated nets, focal drug administration, or reactive case detection (RCD) [10–14]. Led by China’s pioneering use of 1-3-7 in achieving malaria elimination, Myanmar, Thailand, and Vietnam, each adopted the 1-3-7 strategy, while Cambodia and Tanzania are both evaluating 1-3-7 focus investigation pilot projects [10–13, 16].

The definition of a focus area varies by program, from as small as village-level (Myanmar, Thailand) to as large as a county (China) [10, 11, 14]. In pre-elimination settings, where malaria incidence is below 50 cases per 1000 people per year, infections occur in temporal and spatial hotspots, justifying these spatially targeted approaches [17]. All countries reported over 94% compliance to the 1, 3, and 7 day goals respectively, and all used electronic surveillance systems for individual-case-based data collection, data review, and compliance evaluation [7, 10].

The theory of change for digital health surveillance is based on the assumption that enhancing reporting timeliness, completeness, and accuracy leads to efficient management and response with effective interventions [18, 19]. Enabling factors for digital surveillance, identified in 1-3-7 programs, included human resource management and motivation, flexible budget allocation, and strong logistical support [7, 11, 20, 21]. Barriers to digital surveillance included logistical delays, ambiguity in implementation strategies, and lack of political commitment, financial support, or perceived reporting urgency [7, 11, 20, 21].

Despite its use in South-East Asia, this strategy has yet to be studied in southern Africa where malaria transmission heterogeneity, parasite species, technical reporting capacity, and local context differ. For example, Zambia has very low (< 10 cases per 1000 people per year) and very high (> than 3000 cases per 1000 people per year) transmission areas, unlike the Greater Mekong subregion (GMS) where the most intense transmission areas have fewer than 50 cases per 1000 people per year [10]. Simultaneously managing elimination-focused and control strategies requires adept balancing of resources and programmatic prioritization. Further, 98% of malaria infections in Zambia are *Plasmodium falciparum*, while in the six countries in the GMS, many infections are caused by *Plasmodium vivax* [22]. Lastly, mobile phone network coverage differs globally; only 50% of the population on the African continent has access to 4G, in contrast to 96% of the population in the Asia-Pacific region [23]. Digital surveillance systems rely either on internet access or mobile phone networks, the cell phone signal used to send and receive text messages or phone calls. The availability of one of these is central to a functional reporting system.

The Zambia health system is organized centrally, with a national level that develops strategies to be deployed in the 10 provinces, each containing 10–20 districts. Districts oversee the hospitals, health centers and health posts within them, and each of these health facilities delivers centralized and community-based services to their catchment areas. The catchment areas are sub-divided into zones, which are served by individual community health workers (CHW). Surveillance in the Zambian health system, including malaria surveillance, is done at the zonal and health facility level. CHWs document the number of malaria cases in a paper logbook, which they aggregate weekly and report to the health facility. Health facility staff combine these reports with health facility-diagnosed case logbooks to submit a weekly paper report with the total number of cases diagnosed in that health facility each week. This report is then uploaded to the electronic health information system. There historically was no individual case-based reporting nor real-time reporting of malaria cases. However, in 2021, the Zambia National Malaria Elimination Strategic Plan 2022–2026 designated health facilities with annual malaria incidence below 50 cases per 1000 people to be eligible for 1-3-7 focus investigation [24]. Deploying 1-3-7 focus investigation in Zambia required adapting classification schemes to the local malaria epidemiology, designing surveillance systems compatible with the infrastructure, and defining implementation roles and responsibilities [25]. A type two implementation-effectiveness hybrid study was conducted, where the 1-3-7 focus investigation strategy was randomized for a two-year implementation in ten of twenty zones in Choma District, Zambia [25]. Here reported are the results of the mixed-methods implementation science study to evaluate the first year of 1-3-7 focus investigation. Implementation and service outcomes were measured in the zones implementing 1-3-7 focus investigation, factors associated with variability in these outcomes were identified, and select outcomes were compared to those in zones without 1-3-7 focus investigation. Challenges and benefits of 1-3-7 focus investigation were further explored through qualitative interviews to inform potential programmatic scale-up.

## Methods

### Ethics statement

This study was a part of the IRB-approved study, “Magnifying the Utility of Surveillance in Elimination-focused Malaria Operations (MUSEMO)”, which has ethical approval from the Johns Hopkins Bloomberg School of Public Health (Baltimore, Maryland) under IRB no: 00019447 and Macha Research Trust (Macha, Zambia) under IRB no: 0007649. Written informed consent was obtained from adults 18 years and older, and parental permission from parents or guardians of individuals younger than 18 years. Oral assent was obtained from children between 12 and 18 years. Oral consent was obtained for key informant interviews, which collected opinions, experiences, and feedback on the 1-3-7 program but not any personal information.

### Study site and program design

**Fig 1:**
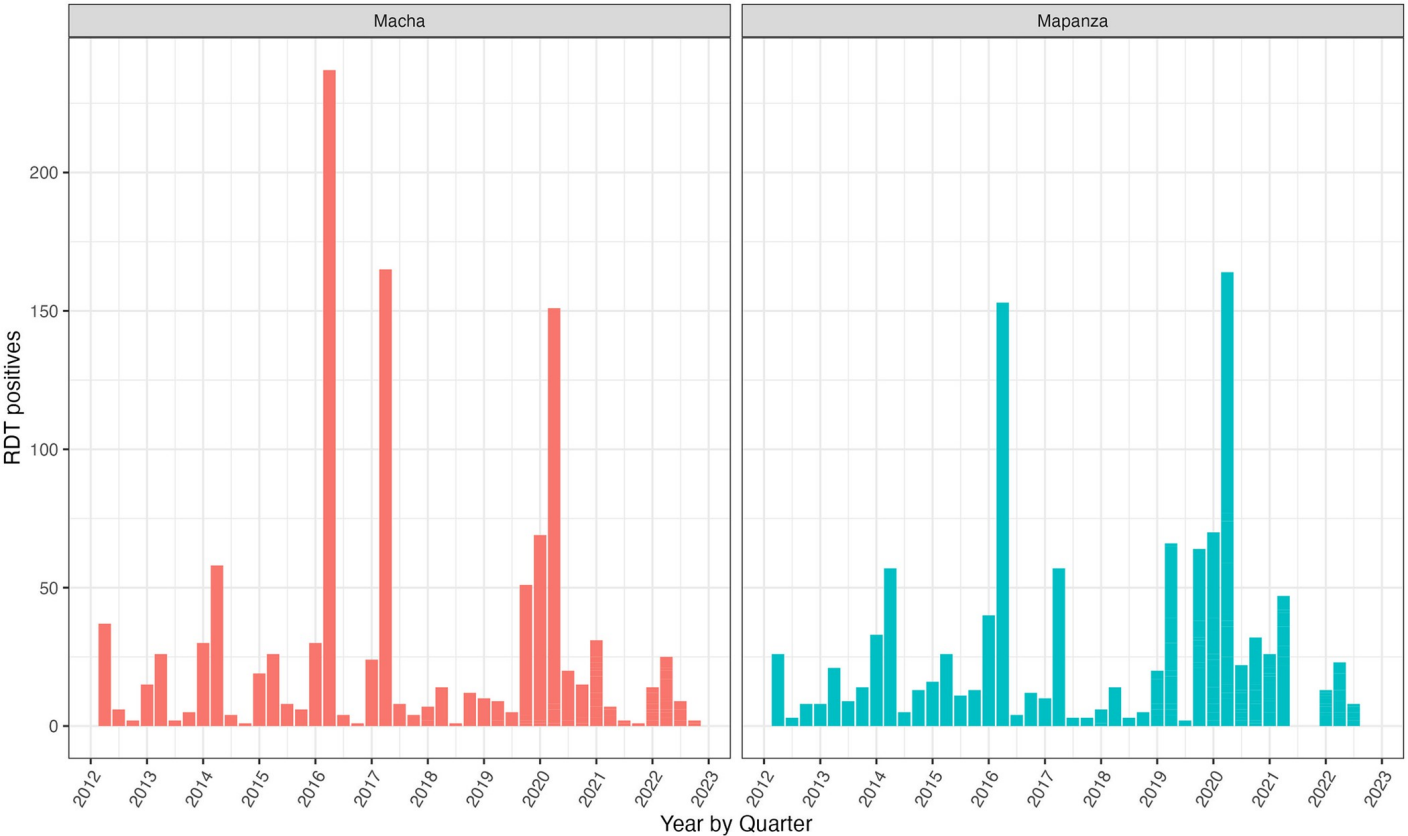
Monthly malaria incidence in study area. Monthly malaria incidence from Macha Hospital and Mapanza Rural Health Center from 2012 to 2022. This data is reported weekly by health facility staff, who submit a report via text message. Seasonal peaks are apparent in the first or second quarter of each year, as is increased incidence in 2016, 2017, and 2020.

This study took place from April 9, 2022 to April 9, 2024 in the catchment areas of Macha Hospital and Mapanza Rural Health Centre, both located in Choma District, Southern Province and serving populations of 13,469 and 15,086 respectively (2021 estimate) [26]. The five-year average annual malaria incidence in Macha and Mapanza were 3.6 and 4.7 cases per 1000 people respectively, with seasonal peaks in April (major) and December (minor) (Fig 1). RCD is the standard intervention conducted in response to passively reported cases in this area, although evidence for RCD impact is varied in moderate to low transmission settings [27]. RCD in this setting includes screening all residents of the index household and in households within 140 meters of the index case by rapid diagnostic test (RDT); those who test positive for malaria are offered treatment with artemether-lumefantrine.

The 1-3-7 focus investigation was adapted in collaboration with the provincial, district, and health facility leadership to address barriers identified in other 1-3-7 focus investigations. The study researchers presented a proposed 1-3-7 focus investigation approach in two sessions with each of the provincial and district malaria officers from the respective health offices. The second session involved an updated proposal incorporating suggestions from the representatives. Following these sessions, the research team presented the approach to a group of health facility staff leadership, including the two nurses-in-charge and five environmental health technicians (EHT), and modifications to the approach were incorporated. To further garner support of the program, the provincial and district officers were engaged monthly in a data use activity that prompted them to review 1-3-7 focus investigation data and subsequently encouraged them to follow up with health facility staff directly responsible for community activities. The research program also supported monthly visits by the district officer to the study area to enhance management presence and facilitate re-trainings as needed to reduce ambiguity on the 1-3-7 strategy.

Specific adaptations of the 1-3-7 focus investigation to the context in southern Zambia included modifications to the definition of a focus area, the definition of an imported versus a local case, transmission strata classification schema, and the technical design of the surveillance system. Focus areas were considered commensurate with zones, the lowest geographic unit in the health system hierarchy. The 1-3-7 strategy was rolled out in April 2022 in 10 of the 20 zones across the two catchment areas. The 10 control zones did not receive the 1-3-7 strategy to facilitate a cluster-level comparison for an effectiveness evaluation, reported elsewhere. These zones continued to implement the RCD intervention without the added surveillance requirements of the 1-3-7 strategy. Cases were reported after diagnosis at a health facility or in the community by a CHW using a malaria RDT. Several case diagnoses at the health facilities were confirmed by microscopy when the health worker was uncertain of the RDT result. Cases were classified in the case investigation step. To be classified as imported, a case must have reported travel outside of their home zone in which at least one night in the preceding two weeks was spent in an area with known malaria transmission. Cases were otherwise classified as local, but these were not further classified as introduced or indigenous. Four transmission categories were created for focus classification. These were cleared transmission (no local cases for last three years), residual transmission (no local case for last calendar year), and active transmission (at least one local case within the last calendar year). Active transmission was further subdivided into active transmission 1 zones (incidence ≥10 cases per 1000 person years over the past year) or active transmission 2 zones (incidence <10 cases per 1000 person years). CHWs were responsible for case notification when diagnosing a case in the community, and facility laboratory technicians and outpatient staff were responsible for case notification when diagnosing a case at a facility. CHWs and EHTs together were responsible for case investigation and focus investigation and response. Focus investigation and response included RCD, reporting any updates to that zone’s focus classification, and reporting recommendations for other interventions, although no additional interventions were deployed during the study period.

**Fig 2:**
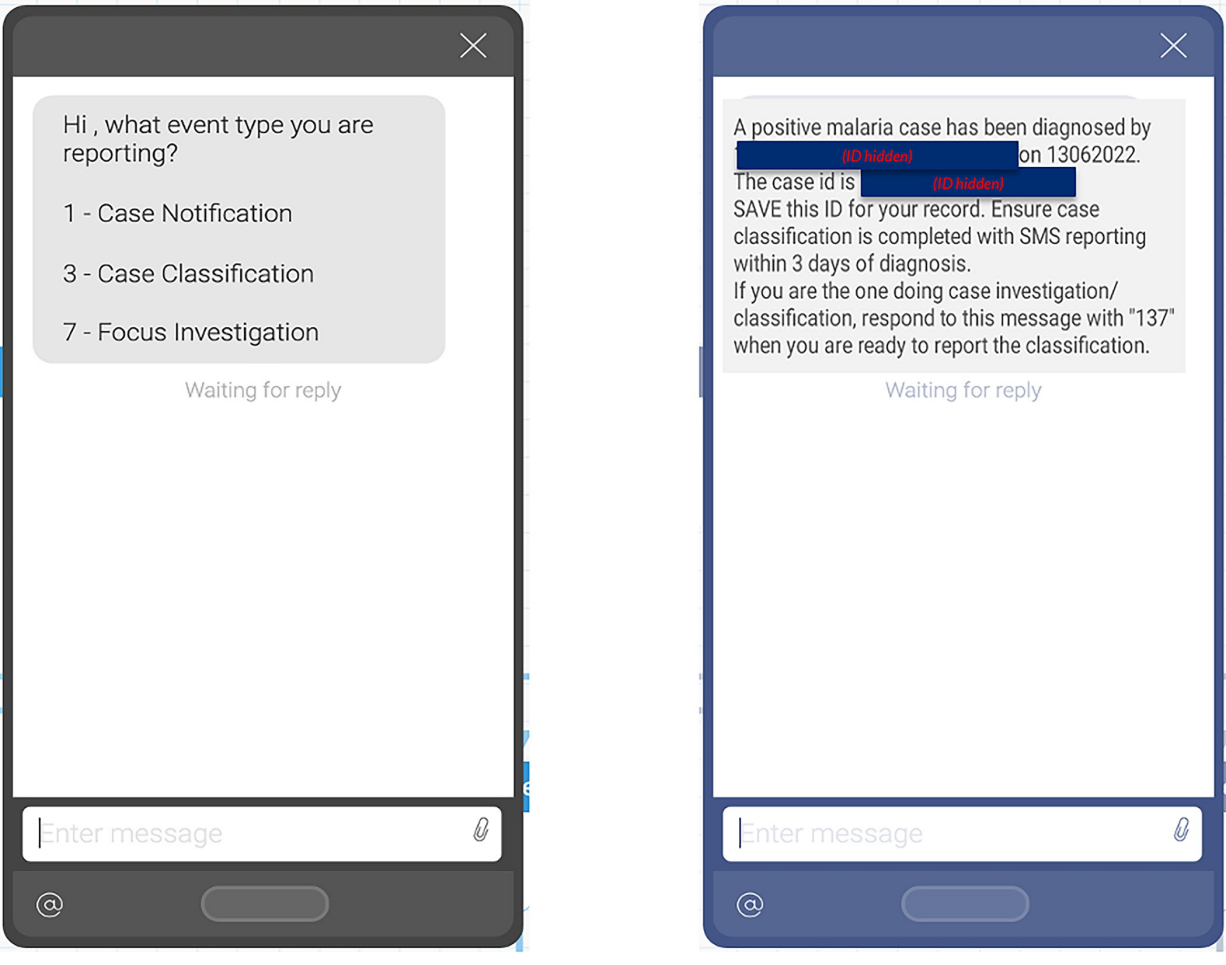
Reporting using the 1-3-7 SMS platform. A reporter texts “137” to +260 ******* to initiate an interactive SMS reporting exchange, with the first message (left) asking the type of event that is being reported. A series of messages then ask for identifying features of the case and, depending on the message type, ask about case travel history (case classification) or features of the focus area of the index case (focus investigation). Each messaging exchange ends with a final message summarizing the exchange (example on right). A copy of the final message is also sent to the facility-based EHT, the facility in-charge, and the district malaria elimination officers.

**Fig 3:**
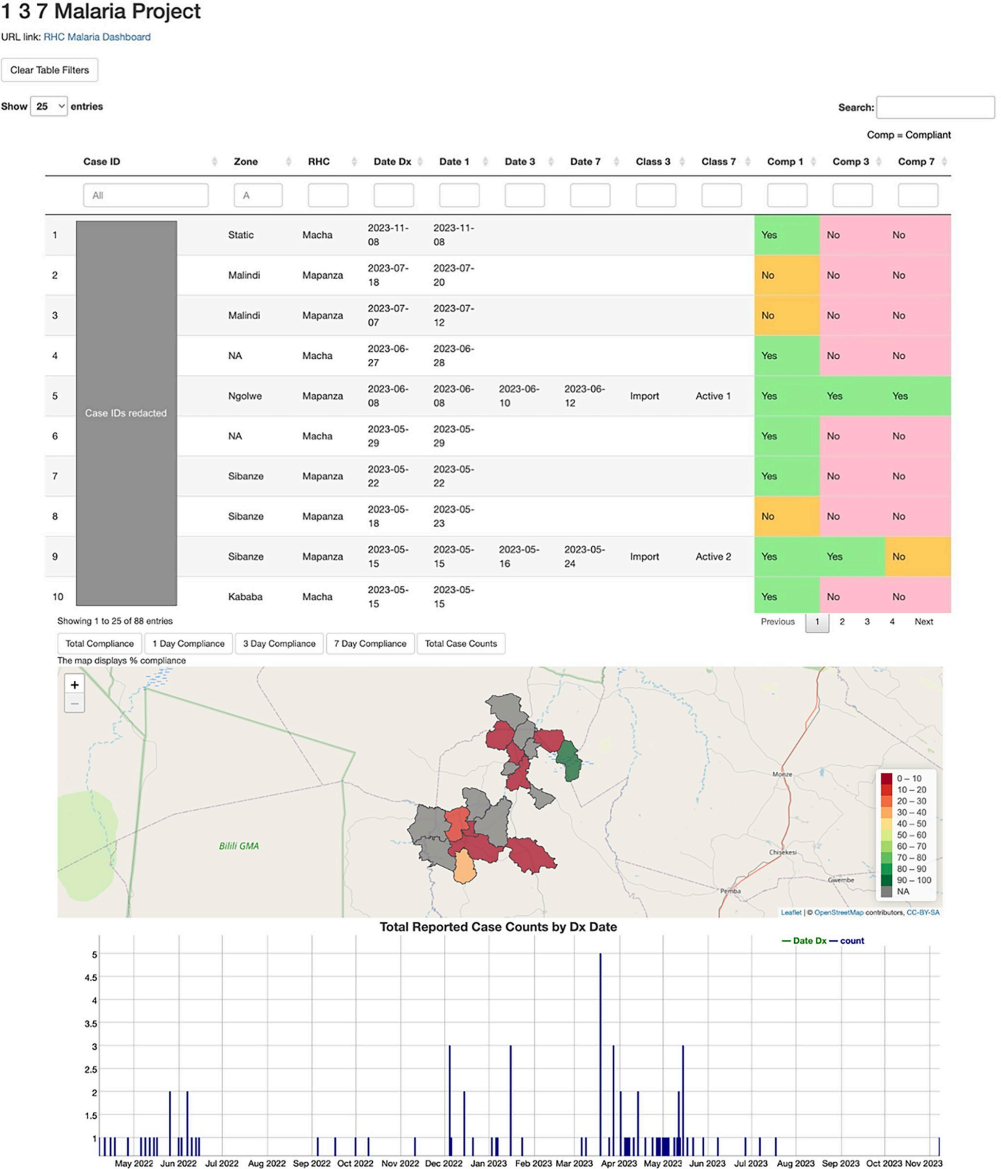
Data presented on the 1-3-7 R-Shiny dashboard. The R-Shiny dashboard displays individual case information, including the date and location of diagnosis, and provides clear visual indicators on each case’s compliance with 1-3-7 strategy in the columns to the far right. Cases are also mapped and reported in aggregate (bottom).

The surveillance system was designed with technologies and hardware that would work best in the local context. Specifically, the system relied on interactive short-messaging-service (SMS, commonly known as text messaging) reporting through the TextIt platform (© 2023 TextIt), rather than Android-based reporting that requires smartphones and internet network access. For each 1-3-7 step, CHWs, lab technicians, nurses or EHTs texted in response to pre-programmed text prompts to collect key data elements. The data elements were sent via integration from TextIt to REDCap (© 2024 Vanderbilt University) and displayed on a web-based dashboard, built using the open-source tool, R-Shiny. R-Shiny can be used to build customized dashboards using R scripts to define indicators and generate data visualizations. For this program, the dashboard displayed indicators of individual case compliance to 1-3-7 goals and mapped incidence in the health facility catchment areas (Figs 2 and 3). Each SMS exchange also triggered an SMS to be sent directly to the CHW and EHT, alerting them to a case in their area and providing instructions for next steps. These notifications sought to provide a sense of urgency, motivation, and a clear directive. All CHWs owned a basic cell phone, eliminating the need for hardware procurement common in smartphone reporting systems. CHWs and EHTs were provided a small mobile credit reimbursement (approximately USD 0.38) for the submission of each SMS notification. For the two trainings conducted on the 1-3-7 focus investigation, CHWs were provided a stipend to reimburse travel expenses to training locations. EHTs were compensated for their participation in data collection for the effectiveness study of the 1-3-7 focus investigation but not for this study. Health workers were otherwise not compensated for participating in 1-3-7 reporting. An identifier was generated for each confirmed malaria case during case notification using the individual’s first name, age, and diagnosis date. This identifier was later used to report subsequent steps in case and focus investigation and link data for individuals on the R-Shiny dashboard.

A research team visited each index-case community seven and 35 days after index case diagnosis. Eligible cases were those that lived within the study area in a non-boarding school setting and were older than six months. These visits included community, household, and individual surveys that collected demographic information, information on bed net usage and travel, as well as information on recent visits from CHWs or health facility staff. The team checked facility registers weekly for new malaria case diagnoses. The research team did not communicate with CHWs or facility staff about the occurrence or timing of cases and conducted community field visits only after CHW field visits were complete. Several additional measures, such as strict communication hierarchies, were in place to minimize the research team’s influence on the 1-3-7 focus investigation.

### Implementation science design

**Fig 4:**
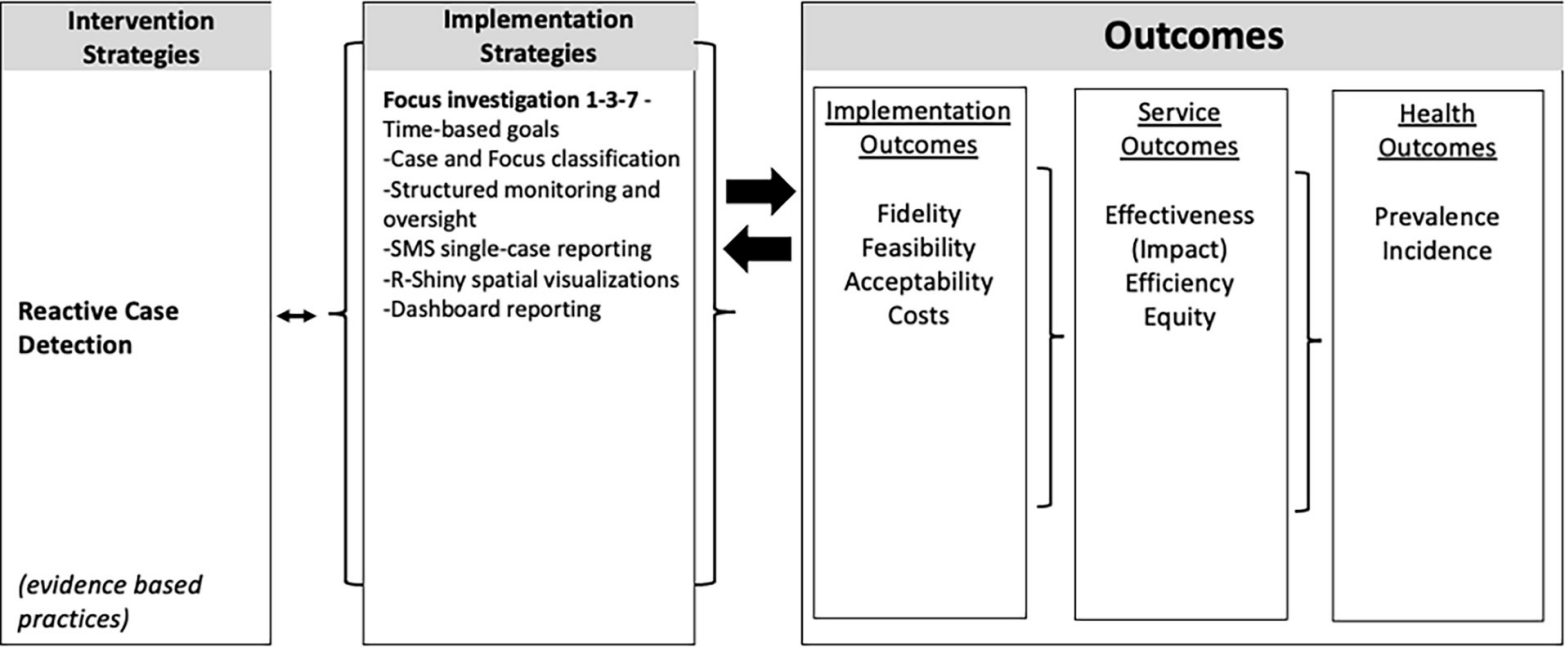
Framework for implementation research. Intervention strategies are proven, evidence-based practice to be implemented. This assumes a specific behavior change intervention is to be selected. Implementation strategies, as well as the context of the implementation, should be described and captured. Outcomes and a method of measurement (as is feasible and applicable) should be defined a priori. Effectiveness and health outcomes should be defined within the effectiveness study as described in Table 1. Figure adapted from Proctor et al, 2009.

Implementation outcomes were latent constructs drawn from a conceptual framework based on the Proctor implementation science model (Fig 4) [28]. The aim of the Proctor implementation science model is to evaluate interventions and implementation strategies, unlike other implementation science models that focus on mapping processes and identify determinants influencing implementation [28]. A health-worker and health-system centered perspective was used. Fidelity, feasibility, acceptability, efficiency, and equity were selected as outcomes given the early-stage of implementing the 1-3-7 focus investigation. Definitions for these outcomes are shown in Table 1, summarized from Proctor et al [29]. Quantitative and qualitative data collection was done both sequentially and concurrently, with the intention that the qualitative data would confirm and expand upon the quantitative data, and together be analyzed through a triangulation and complementarity lens. Quantitative data was collected during the first ten months of the study and was used to design the interviews. The interviews then collected quantitative and qualitative data. Finally, additional quantitative data was collected to better understand findings from the interviews.

**Table 1:** Implementation outcome definitions as defined in Proctor et al, 2011.

Outcome	Definition
Fidelity	To what extent an intervention is implemented as designed
Feasibility	To what extent an intervention can be deployed and utilized successfully given the context
Acceptability	To what extent the intervention is liked, welcomed, and endorsed by its users and recipients
Efficiency	To what extent resources are used relative to intended service or impact outcomes
Equity	To what extent the intervention reaches various populations such that they receive its benefits evenly. Often seen as a function of feasibility, acceptability, and appropriateness

Outcome constructs were measured through pre-defined quantitative indicators from data and metadata collected during community field visits, from the SMS reporting, and from interviews (Table 2). These indicators mapped concepts of the implementation constructs to their definitions, which also captured constructs measuring barriers and enablers identified in earlier evaluations. For example, acceptability indicators may measure human resource motivation and political commitment (Table 2, A1 –A12), feasibility indicators measure logistical issues (Fs4, Fs8 –Fs10) and implementation strategy ambiguity (Fs5, Fs7), and fidelity indicators approximate the perceived urgency of reporting through completion rates (Fd1 –Fd6). Most indicators measured outcomes in the ten zones implementing 1-3-7 focus investigation, although several fidelity and efficiency indicators were also measured in the ten control zones in the effectiveness study. Feasibility and acceptability were also measured qualitatively through open-ended questions in semi-structured interviews [30].

**Table 2:** Indicators used to measure outcome constructs.

Indicator No.	Indicator	Indicator definition	Data source
** **	**Fidelity**	Compliance to 1-3-7 as designed	** **
Fd0	Eligible cases reported digitally	Cases that are reported digitally	TextIt
Fd1	Day 1 Compliant	Cases with on-time notification/ Total cases	TextIt, REDCap
Fd2	Day 3 Compliant	Cases with on-time classification/ Total cases	TextIt, REDCap
Fd3	Day 7 Compliant	Focus areas with on-time investigation/ Total cases	TextIt, REDCap
Fd4	Time to notification	Notification date—diagnosis date	TextIt, REDCap
Fd5	Time to classification	Classification date—diagnosis date	TextIt, REDCap
Fd6	Time to investigation	Investigation date—diagnosis date	TextIt, REDCap
Fd7	RCD done	At least one household within 140 m is marked as having had follow up after index case	REDCap
Fd8	RCD completion	Visited within 140 / Total households within 140	REDCap
Fd9	RCD error	Households visited outside of 140 m/ Total households visited	REDCap
Fd10	Zone accuracy	Accurately identified zones or villages mappable to zone / total patients RDT positive at facility	Registers and REDCap
** **	**Efficiency**	Efficiency of focus investigation in detecting cases	** **
Ef1	Index HH RDT prevalence	Household RDT positives/Household members	REDCap
Ef2	Extra-index RDT prevalence	RDT positives in households outside of index/Household members in households outside of index	REDCap
Ef3	Proportion RDT-positive in Index	RDT positives in index / RDT positives in focus	REDCap
Ef4	Proportion RDT-positive outside Index	RDT positives outside of index / RDT positives in focus	RedCAP
** **	**Equity**	Reach of 1-3-7 program across different demographic groups	** **
Eq1	Proportion missed	Missed individuals/ total individuals on registers	REDCap
Eq2	Proportion community diagnosed cases	# cases diagnosed in community / total diagnosed cases	REDCap
** **	**Feasibility**	Ability to carry out tasks and processes required by 1-3-7	** **
Fs1	TextIt Completion	% of notifications that had the full chain of messages completed (Each step, eg "1" step, is evaluated as an independent notification)	TextIt
Fs2	TextIt Length	Total time between first and last message of complete SMS conversations	TextIt, Interview
Fs3	1-3-7 Initiation	Percent of index cases for which 1-3-7 was initiated	TextIt, Case tracking with study team
Fs4	SMS issues	How frequently users experienced issues with network, phone, talk time that prevented reporting	Interview
Fs5	SMS ease	Likert score of ease of use	Interview
Fs6	Network availability	Strength of cell signal across areas where 1-3-7 reporting was done	Network on-ground survey
Fs7	R-Shiny ease	Likert score of ease of use	Interview
Fs8	R-Shiny issues	How frequently users experienced issues with network, phone, talk time that prevented reporting	Interview
Fs9	ID errors	Percent of index cases that were tied to at least one id error that prevented linkage of messaging	REDCap and project data
Fs10	ID duplication	Count of times that a unique ID was duplicated and used across two different cases	REDCap and project data
Fs11	Morphology correctness	Percent of mosquitoes classified that are classified accurately to genus level	Paper forms submitted by CHWs, Ento team data
Fs12	Errors	Count of SMS sent that were not automatically processed and linked to cases due to typos or issues with server syncing	Aggregated and estimated by SMS data manager
** **	**Acceptability**	Extend to which program is liked and welcomed	** **
A1	1-3-7 approval	Level (Likert ranked) of approval of 1-3-7 program.	Interview
A2	1-3-7 appeal	Level (Likert ranked) of appeal of 1-3-7 program.	Interview
A3	1-3-7 liked	Level (Likert ranked) of like of 1-3-7 program.	Interview
A4	1-3-7 welcomed	Level (Likert ranked) of welcomeness of 1-3-7 program.	Interview
A5	SMS approval	Level (Likert ranked) of approval of SMS reporting.	Interview
A6	SMS appeal	Level (Likert ranked) of appeal of SMS reporting.	Interview
A7	SMS liked	Level (Likert ranked) of like of SMS reporting.	Interview
A8	SMS welcomed	Level (Likert ranked) of welcomeness of SMS reporting.	Interview
A9	R-Shiny approval	Level (Likert ranked) of approval of R-Shiny dashboards.	Interview
A10	R-Shiny appeal	Level (Likert ranked) of appeal of R-Shiny dashboards.	Interview
A11	R-Shiny liked	Level (Likert ranked) of like of R-Shiny dashboards.	Interview
A12	R-Shiny welcomed	Level (Likert ranked) of welcomeness of R-Shiny dashboards.	Interview

Outcome constructs (e.g. fidelity) are mapped to multiple indicators. Indicators have specific definitions that can be mapped to data elements that come from the specified data source.

After qualitative data collection, cell phone network strength was identified as an important feasibility indicator and a network strength survey was conducted in 33 locations in the study area, identified by CHWs and EHTs as the locations from which they send SMS to the 1-3-7 reporting system. These were most commonly CHW homes and health facility offices. In each location, network strength in decibels per milliwatt (dBM) was measured between 11 am and 3 pm. Six measurements, each five minutes from the last, were taken at each location on the research team’s device and the device used by the CHW or EHT. The correlation between reporting time and network strength was examined. Case reporting audits comparing facility RDT registers, community RDT registers, and study data were conducted routinely by the study team to assess when and why cases were missed by the 1-3-7 focus investigation.

Descriptive analyses were conducted for each quantitative indicator. Chi-squared tests, analysis of variance (ANOVA) tests, and t-tests were used to compare distributions of key indicators across different case types (imported versus local), focus types (active versus non-active), and distance from health facility, to identify effect modifiers of implementation success. Chi-squared tests were also used to compare distributions of fidelity and equity indicators across intervention and control zones.

All twenty-four individuals involved in the 1-3-7 program, who were either CHWs, lab technicians, EHTs, or district and provincial managers, were identified and invited to participate in the interviews. Final participation was determined by availability and whether individuals agreed to participate. Each interview followed a standard template based on the interviewee’s role. Likert-rank questions from the validated AIM tool were used to assess feasibility and acceptability of the SMS notification system, the web-based app, and the 1-3-7 focus investigation as a whole [30]. Open-ended questions allowed participants to expand upon the topics discussed and directly speak to barriers and facilitators [30]. Each interview consisted of one interviewer and two notetakers, and a master transcript was compiled in a debrief session immediately following each interview. Notes were typed or taken by hand, and no recording device was used. Interview team members had working relationships with the interviewees through the 1-3-7 program but introduced interviews as an opportunity for free expression of thoughts, opinions, and feelings on the 1-3-7 program. Interviews were conducted in a private space at the health facility, such as an office or low-trafficked common area. Inductive coding with a framework approach was used to conduct thematic interview analysis of the open-ended survey questions. Codes were organized into four pre-defined themes (barriers, benefits, enablers, and contextual factors) to inform feasibility, acceptability, and potential program expansion. Two coders independently read all interview transcripts and identified preliminary codes within the a priori themes. The coders discussed the codes to form a consensus set of codes before re-coding all interviews. The authors validated the implementation and mixed-methods study design against the Standards for Reporting Implementation Studies (StaRI), and Standards for Reporting Qualitative Research (SRQR) [31–33].

## Results

Between April 2022 and June 2023 there were 62 passively reported malaria cases in areas implementing the 1-3-7 focus investigation, and 41 were eligible for 1-3-7 follow-up. Cases were ineligible if living at a boarding school (9), being a secondary case diagnosed concurrently with the index case (8), living outside the study area (1), being a study team member (1), or reason unknown (2). Most, 71.4%, of the 41 eligible index cases were diagnosed at the health centers and the rest were community-diagnosed by CHWs. Of these cases, 34 (83%) were notified, 29 (71%) were classified, and 13 (32%) focus areas were investigated and followed-up. Eighteen (44%) cases were classified as local and 11 (27%) as imported. At the start of 1-3-7 implementation, one zone had an incidence above 10 cases per 1000 people per year and was classified as active transmission 1 (high), and nine zones were active transmission 2 (low). No zones were classified as residual or cleared transmission. By the end of the first year, one zone upgraded from active transmission 2 to active transmission 1, a higher transmission classification, but otherwise no classifications changed. During this same period, 34 cases were passively reported in areas implementing standard RCD, 27 of which would have been eligible for 1-3-7 focus investigation had they resided in the focus investigation area.

The study team visited 1595 and 1285 household members at the first and second visits in the 1-3-7 focus investigation areas. The participants were 55% female and the median age was 16 years (interquartile range [IQR]: 8,30). These individuals resided in 312 households across 10 zones. For the semi-structured interviews, 17 individuals were interviewed (Table 3), three declined to participate, one was not available, and three were not interviewed as content saturation was reached.

**Table 3:** Interview eligibility and participation by role.

Role name and description	Total	Total participating
Data collectors, type 1	13	9
Community health workers who report through the 1-3-7 SMS system		
Data collector, type 2	13*	6
Facility-based lab staff who report through the 1-3-7 SMS system		
Data reviewer and decision maker, Level I	9	5
Environmental Health Technicians who manage of CHWs and lab staff using the R-shiny dashboard		
Data reviewer and decision maker, Level II	9	5
Health officials at the district and provincial level who oversee these teams and may use the data for larger scale decision-making		

Overview of individuals in each job role participating in the 1-3-7 focus investigation and the selection of these individuals who participated in interviews.

* Due to turnover in the outpatient departments, 21 type 2 data collectors participated in the 1-3-7 focus investigation but at the time of interviewing only 13 were currently working in that position.

### Fidelity

**Fig 5:**
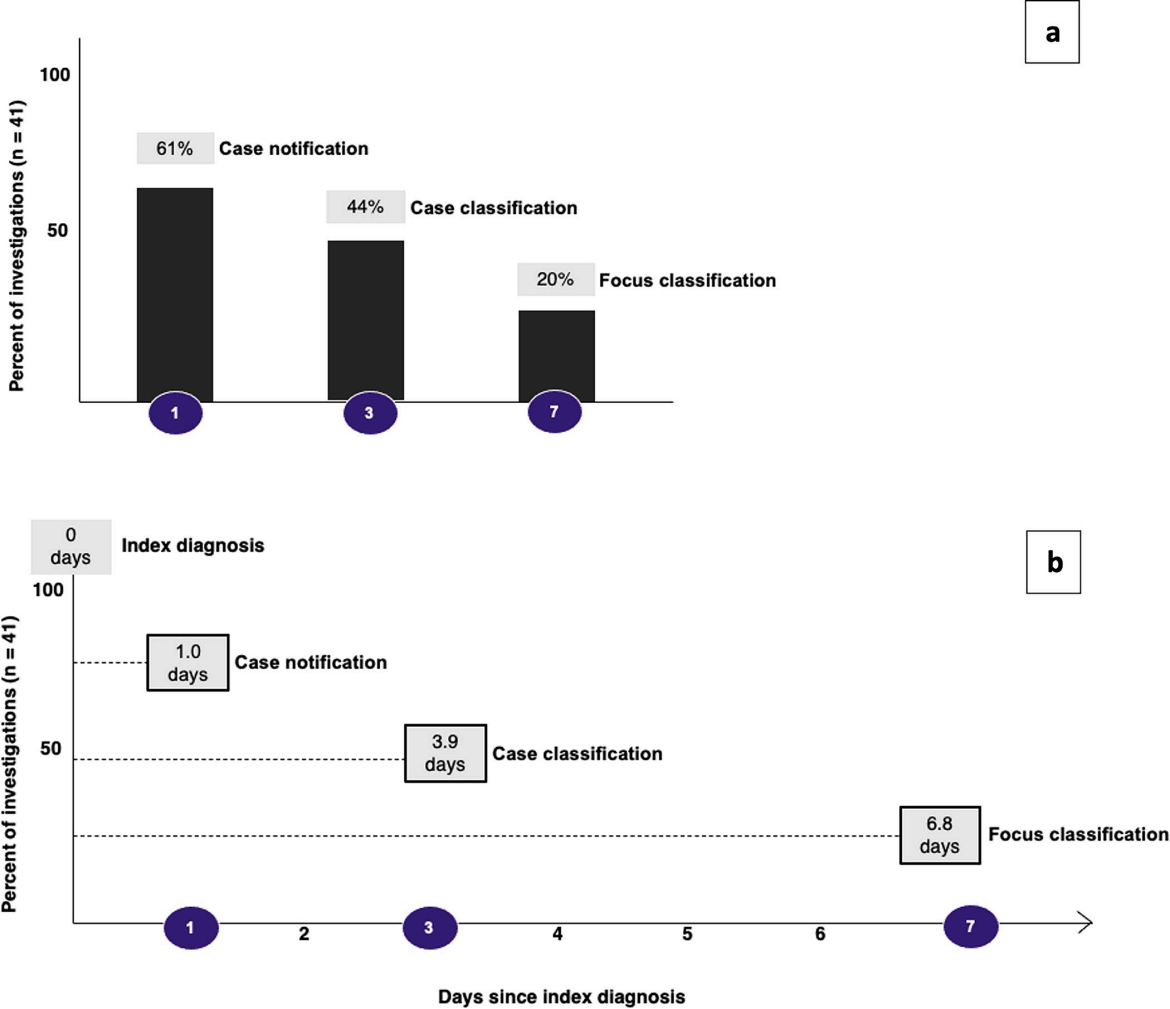
Fidelity and average time to reporting of case notification, case classification, and focus classification. Panel a (top) shows fidelity measured as on-time completion of case notification, case classification, and focus classification. 61% of case notifications, 44% of case classifications, and 20% of focus classifications were completed on time, within one, three, and seven days of index diagnosis respectively. These were fully compliant to steps on days 1, 3, and 7. Panel b shows the time taken for the reporting of case notification, case classification and focus classification. On average, case notification occurred 1.0 days after index diagnosis, case classification occurred 3.9 days after index diagnosis, and focus classification occurred 6.8 days after index diagnosis. This includes all notifications that were complete, averaging time to completion across all.

Of the 41 eligible cases, 61%, 44%, and 20% were compliant with steps 1, 3, and 7, completing tasks by the specified deadline. On average, notification occurred 1.0 day after diagnosis, classification occurred 3.9 days after diagnosis, and focus investigation occurred 6.8 days after diagnosis (Fig 5).

**Fig 6:**
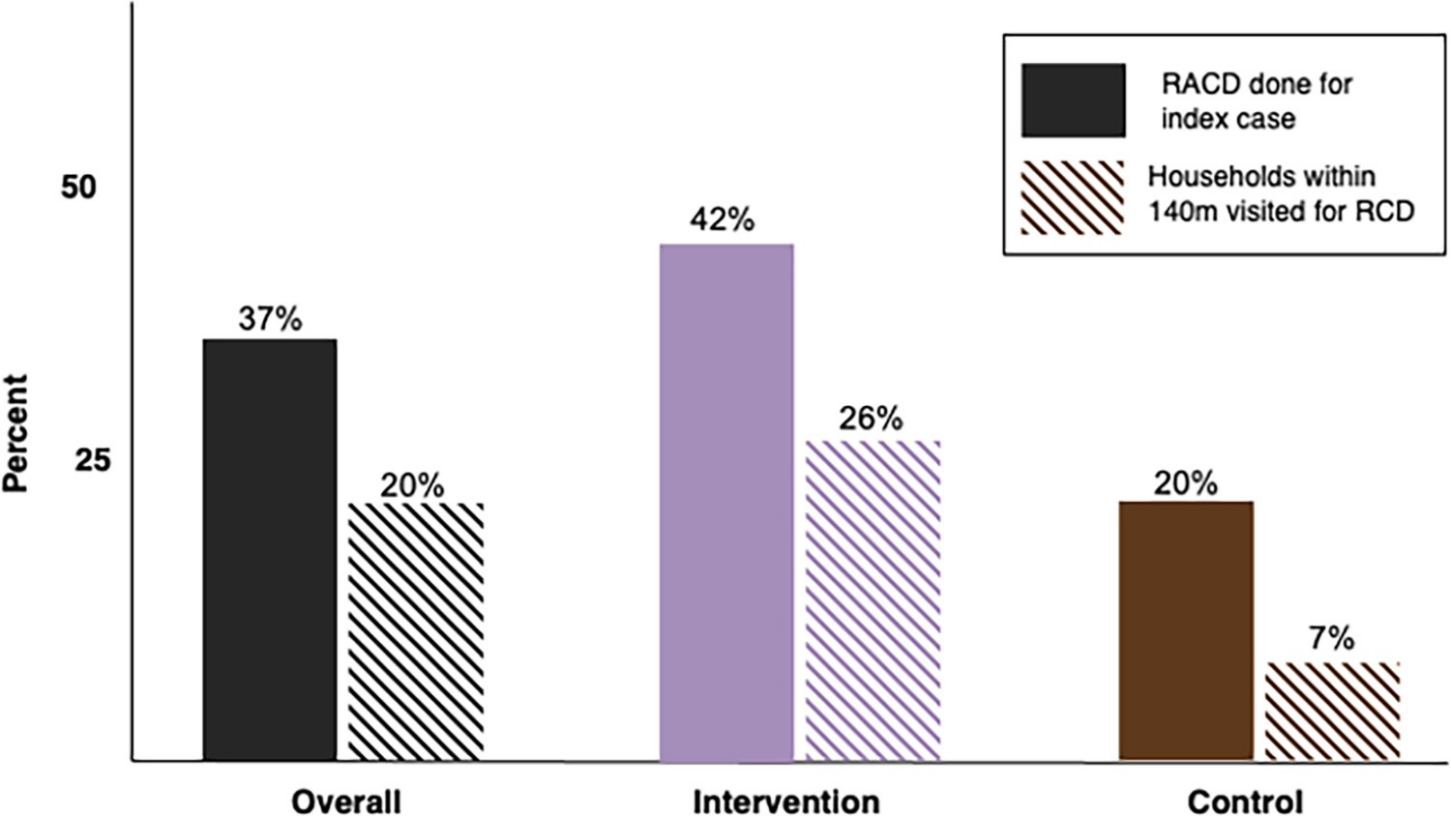
Reactive case detection completion and coverage by arm. Reactive case detection (RCD) completion (solid) and coverage (striped) was higher in intervention areas implementing 1-3-7. RCD was considered complete if at least one house neighboring the index household was visited for RCD. Coverage was defined as the percentage of eligible households that were visited for RCD. Households within 140 meters of the index household were eligible. 1-3-7 focus investigation was associated with higher completion (p-value <0.01) and coverage (p-value < 0.01).

RCD completion and household coverage in 1-3-7 zones were both low. RCD was completed for only 42% of index cases and only 20% of eligible houses were visited by CHWs for RCD. However, completion and coverage were even lower in control zones where completion was 26% and only 7% of eligible houses were visited (Fig 6). The odds of RCD completion in 1-3-7 clusters were 2.7 times than the odds in control clusters, though this difference was not statistically significant (odds ratio [OR]: 2.67, 95% confidence interval [CI]: [0.43, 29.89], p-value = 0.29). The odds of a household visit for RCD in intervention clusters were 4.2 times the odds in control clusters (OR: 4.15, 95% CI: [1.51, 14.31], p-value = 0.002). Thirty-six percent of households visited in intervention areas fell outside the 140-meter radius.

**Fig 7:**
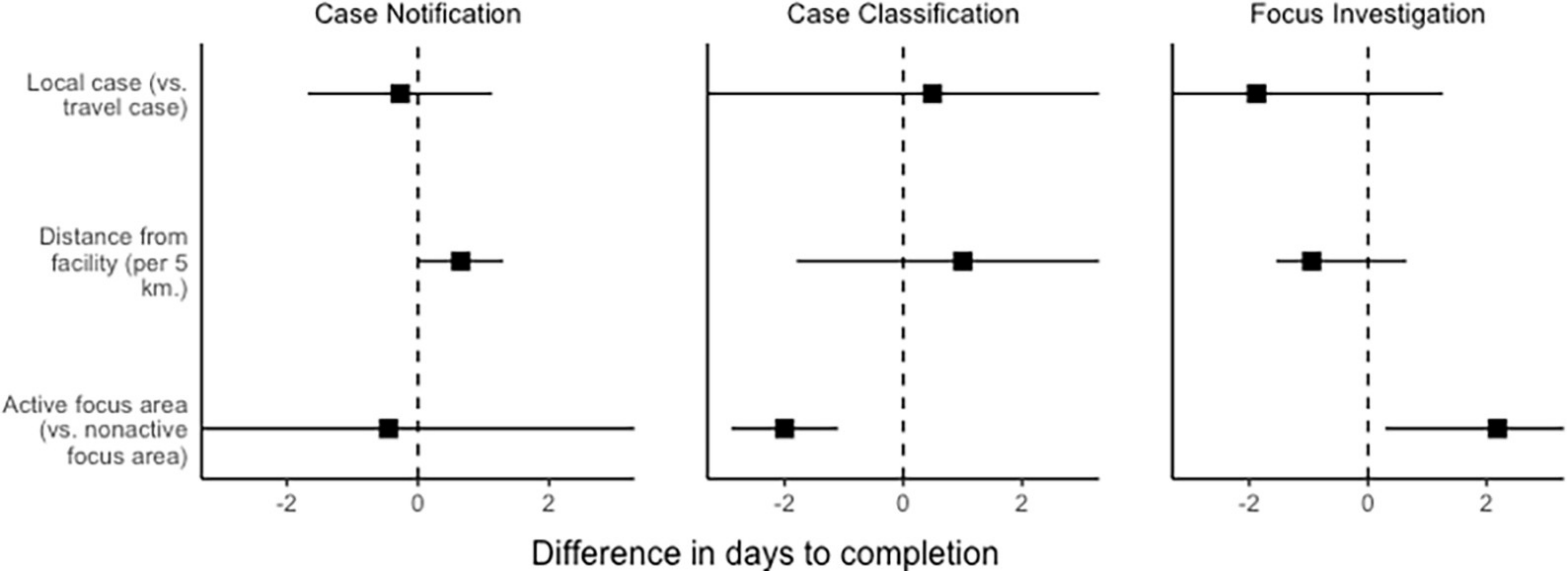
Modifiers of fidelity. Potential modifiers of fidelity were assessed by examining their univariate association with time to completing case notification, case classification, and focus investigation. Comparisons were done using a t-test.

Distance from the household to a health facility and CHW-reported focus classification may modify the effect of 1-3-7 reporting on RCD timeliness and completion (Fig 7). Time to case notification was longer for households farther from heath facilities. Areas where CHWs classified transmission as active (versus non-active) had shorter time to case classification (absolute difference 2 days, 95% CI: [1.10, 2.90], p-value <0.001) but longer time to focus investigation (2.2 days longer, 95% CI: [0.29, 4.08], p-value = 0.02). There were no associations between case classification (local vs imported) and 1-3-7 reporting or follow-up time.

In thematic analysis of the key informant interviews, sustainability emerged as a potential barrier to program fidelity. CHWs and facility-based health workers were compensated with mobile phone credit, also known as talk time, and expressed that this incentive must continue:

“If there was no money, no it wouldn’t go on. Because it requires talk time. They are not free messages. Nurses in [Outpatient Department] (OPD) are overwhelmed, so if there was no motivation then I don’t think it would carry on.

• In-charge at facility, 28062023_Incharge_9

"We will work but we will have challenges in sending messages because we would have to buy talk time with our own money… it would be hard."

• 28062023_CHW_10

### Feasibility

**Fig 8:**
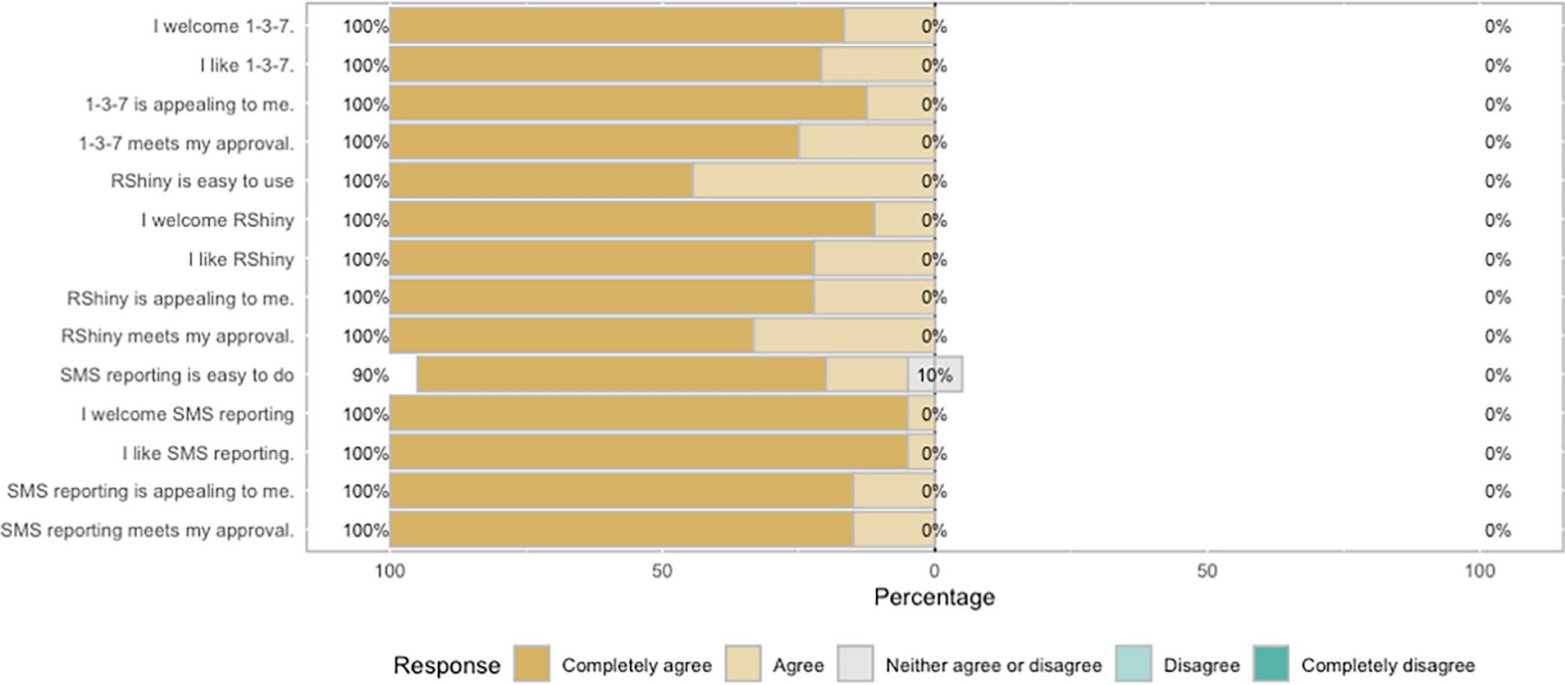
Feasibility and acceptability of the 1-3-7 focus investigation and the R-Shiny dashboard. Feasibility and acceptability of the 1-3-7 focus investigation and the R-Shiny dashboard was high as reported via Likert-score ranked responses.

**Fig 9:**
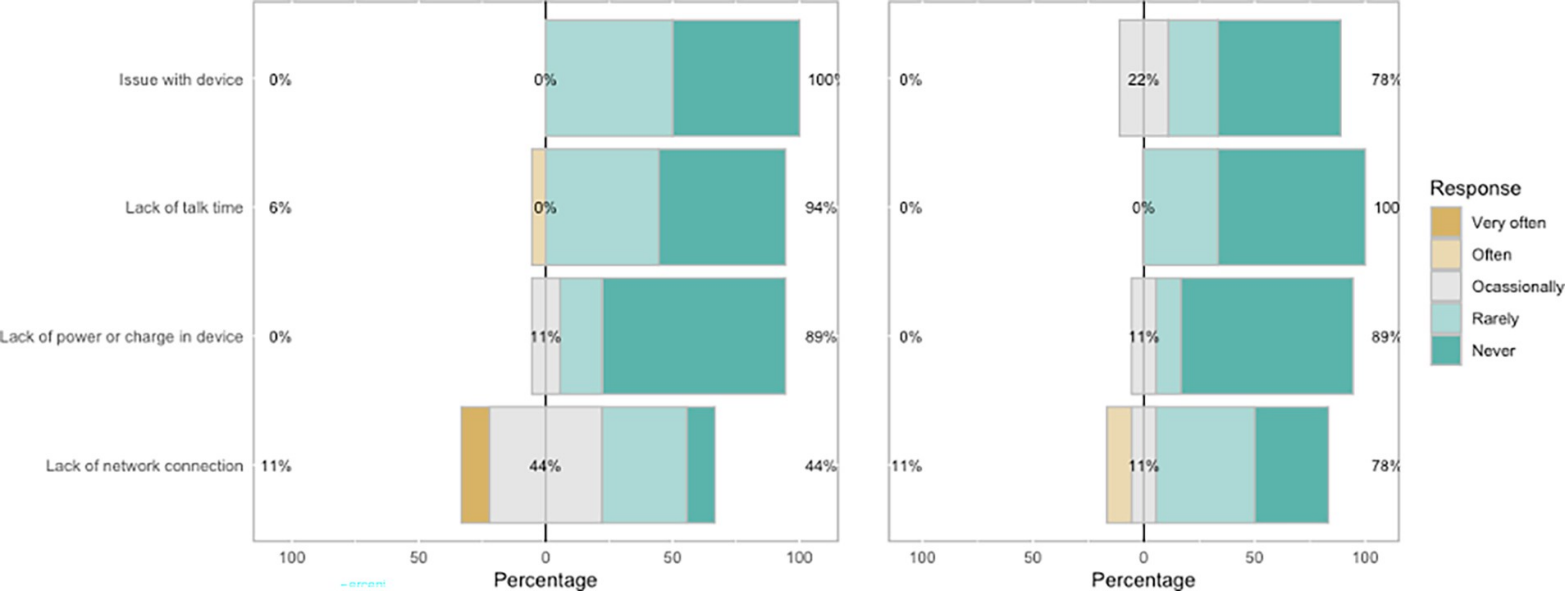
Logistical issues contributing to feasibility. Feasibility ranked order (very often to often) frequency of experiencing logistical issues for the 1-3-7 focus investigation (left) and the R-Shiny dashboard (right) identified challenges with network connection.

Thirty four of 41 eligible cases (82.9%) had SMS notifications initiated. Eighty four percent of interactions with the messaging platform were completed, and the average duration of reporting–the time to complete a full “conversation” as measured by SMS time stamps–was 48 minutes. The average perceived time of interaction, as reported by survey respondents, was 26 minutes. Ninety percent of survey respondents agreed or completely agreed that both 1-3-7 SMS messaging and the R-Shiny application were easy to use (Fig 8). Logistical issues with hardware, power, or network were infrequent, although most users reported logistical issues at least occasionally with SMS reporting (72%) or the R-Shiny application (55%) (Fig 9). Mobile phone network-related delays were the most commonly reported logistical challenge. Average measured network strength across the area was good at -85.2 dBm, but ranged from very good to poor (range: -71.3, -107.3). Of the 33 network strength locations sampled, 14 had consistently poor (< -100 dBM) network connectivity as averaged over six readings, and 26 had at least one poor reading. Network strength varied by mobile network provider (e.g., Airtel, Zamtel, MTN). There was a moderate inverse correlation (R-squared = 0.35) between network strength and time to complete reporting. Other perceived logistical issues were delays in the preprogrammed SMS response time and busyness with other tasks.

The case identifier format (including the first name, age, and date of diagnosis) worked well to uniquely identify cases–no two individual cases had duplicate numbers. Typographical errors and inconsistent spelling in case identifiers resulted in errors in messages failing to link but this only occurred six of 88 times and each was resolved by resending the message. There were four other errors in messages due to syncing across reporting and data visualization platforms.

Reporting failures, technical issues, and the inability to locate the home of index cases during follow-up emerged as barriers to feasibility during thematic analysis of health worker interviews. Reporting failures and delays arose predominantly due to weak network connectivity but also from job-based challenges like busyness or competing responsibilities. At times, workers were frustrated and delayed by slow and inconsistent pre-programmed replies in SMS conversations:

"Sometimes it takes hours. At one point, I started around 15:00 and I received a response [from the SMS platform] around 16:00. When I repl[ied], again it [would] take time. But sometimes, just in 5 minutes it will be done."

• Outpatient department staff, 28062023_OPD_7

Technical issues included typographical errors in SMS messages, TextIt android aggregator bugs, challenges with reporter registration with the TextIt system, and access issues to the R-Shiny dashboard. Finding all passively detected cases was another challenge as many cases were individuals who lived outside the catchment area. At their time of diagnosis, some appropriately identified themselves as visitors while others hid their visitor status to avoid a higher service fee.

“Those who have come to this catchment for services and lie and give the wrong address [because] they want to be seen as coming from within.”

• Community Health Worker, 2706202_CHW_2

CHWs and EHTs suggested adding information in the notification reporting that would facilitate locating index cases, like village name, phone number, or name of child’s guardian. Delays also resulted from stockouts of artemisinin combination therapy and low health center staffing on weekends.

### Acceptability

The SMS reporting, R-Shiny dashboard, and 1-3-7 focus investigation program were broadly accepted. All CHWs, EHTs, and district officers agreed or fully agreed with statements of approval, appeal, liking, and welcoming each of the three components (Fig 8). However, barriers to community acceptance arose in thematic interview analysis. Health workers discussed that communities may distrust or avoid the health system and cited one community that rejected RCD. They mentioned individuals who feared having a blood specimen collected or did not trust health workers’ intentions when conducting the malaria rapid diagnostic testing.

“Some refuse because of the mark of the beast…they think it might be rituals or satanism, devil worship.”

• Community Health Worker, 28062023_CHW_12

These themes arose infrequently but suggest that the program acceptability is not universal.

On the contrary, there were several enabling factors of 1-3-7 focus investigation that provided evidence of its acceptability. Health workers had a broadly positive attitude and demonstrated dedication to their work. Widely held was the belief that doing such monitoring was “right” and that it contributed towards the reduction of malaria. Community health workers demonstrated commitment, resourcefulness, and motivation in describing how they overcame obstacles to completing tasks. They expressed that their inclusion in the program brought them a sense of both validation and appreciation.

“1-3-7 has enhanced that the community always seek help from those who are CHWs. We are more visible. We have quicker response when there is a malaria case.”

• 06292023_CHW_13

The perceived benefits further suggested the 1-3-7 focus investigation was accepted. Health workers cited improvements to communication networks and processes, including that 1-3-7 focus investigation streamlined and enhanced reporting and increased transparency, encouraging adherence to follow-up tasks. It was believed to increase efficiency and accelerate follow up without interfering with other work.

“It is a good channel, it is quick. You are able to notify those responsible for following up the cases…you are able to quickly send, even if they are far.”

• Lab staff, 27062023 _Lab_1

Two health workers even requested that the 1-3-7 program provide more feedback or reminders. A frequently discussed benefit was that both the SMS messages and the R-Shiny dashboard made information more accessible, increased information sharing, and increased use of information in decision making. Health workers believed this information better informed them of malaria incidence and transmission in their areas.

"I know that there is work to be done in my zone…because [of] the [1–3–7] notification.”

• Community Health Worker, CHW: 27062023 _CHW_3

"R-Shiny allows us Data CHWs to review performance from zones. We meet every Wednesday and Friday."

• Data (lead) Community Health Worker, 27062023_CHW4

Accuracy was also cited as a benefit–workers believed it led to a reduction in errors and provided an opportunity to cross-check other data sources. Health workers saw 1-3-7 notifications as a job aide:

“I like the program because it helps us and guides us at every step. It tells us what to do."

• Community Health Worker, 28062023_CHW_10

Senior level staff at the district and provincial level also saw the R-Shiny dashboard as a job-aide that made clear which areas need additional support:

"I am able to see what is happening there, while I am here… the fact that I am able to see if they did the response on time—I like that. And I follow that. If they didn’t do it, I try to find out why… it’s user friendly; even when someone hasn’t really explained [it], you are able to tell what is happening.”

• 29062023_DistrictOfficer_15

Perhaps because there was low day 7 fidelity, senior health staff expressed wanting the R-Shiny dashboard to show them what is done after a case was notified.

“I would want it to stay, because there is good communication when we find a case. The EHT is notified as well as CHWs, so we are also able to follow up how many positives we have, except we don’t get the feedback for whatever goes on after the follow-ups are done…. as nurses it is best that we get some feedback on what goes on, what happens in the field.”

• 28062023_NurseIncharge_9

### Efficiency

Throughout the study period and across both intervention and control areas, only two malaria RDT positive individuals were detected during RCD. Efficiency indicators for RDT positive follow-up cases were therefore not calculated.

### Equity

At the 7-day and 35-day visits, 7.6% and 20.6% of household members in 1-3-7 focus investigation areas were missed. These missed individuals were more likely to be male (16% and 12% missingness in male and female participants, respectively) and under 1 or over 16 years (38% and 18% missingness respectively, as compared to 10% missingness in 1 to 16 year-olds). There was no difference across missingness by case classification, focus classification, or distance from health facility. Health workers reported that individuals who are employed or working in their fields were more likely to be missed.

“Those who work…You find that in that area, maybe 35% are not around for example. Some they are workers, some have business, some at the market. When you go there you find the house is closed”

• Environmental Health Technician, 27062023_EHT6

Household distance from health centers emerged as a barrier to feasibility and also a barrier to equity in the 1-3-7 focus investigation program. An EHT spoke on the challenges:

“Apart from network, I think transport. Once the [Community Health Workers] are called on their phone [to come test someone in the community] but the distance is far. Some have bicycles but some don’t have.”

• Environmental Health Technician, 27062023_EHT6

## Discussion

This work measured implementation outcomes of 1-3-7 focus investigation in ten zones in two low-transmission catchment areas in Choma District in southern Zambia, documenting broad acceptance and feasibility but also gaps in fidelity and equity. Most of the 62 passively reported cases were diagnosed at the health center, due in part to the low RDT stock to share with CHWs for community testing in these low transmission areas. Fidelity to the intervention was moderate, with only 61% of cases being notified on time. RCD was done in 41% of the eligible areas, reaching only 26% of eligible houses, which, while low, was double the coverage of RCD in areas not using 1-3-7 focus investigation [17]. There were gaps in the equity of 1-3-7 focus investigation in reaching non-care-seeking individuals, teenage and adult males, and those in communities farther from health facilities. The same groups of hard-to-reach individuals were missed in other countries implementing 1-3-7 focus investigation who typically are at higher risk of malaria [11, 21, 34]. Importantly, the 1-3-7 focus investigation was feasible and widely accepted by the health facility, district, and provincial staff. Plans to scale-up or adapt 1-3-7 focus investigation in similar low malaria transmission settings would likely be met with enthusiasm but should be sensitive to context-specific limitations to fidelity and feasibility. Scaling-up 1-3-7 focus investigation must also consider how to sustainably maximize penetration and adoption with appropriate resource planning.

Several recommendations emerged from this work and key recommendations are listed in Table 4. First, programs must consider challenges to fidelity and which are readily solvable. Fidelity may improve with stronger management and oversight but the lower fidelity of this 1-3-7 focus investigation (61%) may be partially attributed to barriers in sending SMS messages. Even in 1-3-7 focus investigation programs in Thailand, Myanmar, and Vietnam, poor mobile phone network coverage affected reporting time and completion [10, 11, 20]. Weak mobile phone network coverage should not preclude 1-3-7 focus investigation deployment but reporting delays and failures will occur, lower fidelity should be anticipated, and adoption should be monitored. Surprisingly, hardware feasibility issues ubiquitous in other 1-3-7 focus investigation and digital surveillance systems were not identified in this study [20, 35, 36]. This implementation used SMS reporting, which minimized hardware procurement and reliance on internet connection (the latter being distinct from and scarcer than mobile phone network connectivity). While well-funded, short-term surveillance systems in sub-Saharan Africa are often designed with smartphone-based reporting, 1-3-7 deployment should consider SMS reporting to avoid hardware issues and minimize cost and increase sustainability.

**Table 4:** Key recommendations for the scale-up of 1-3-7.

Finding	Recommendations
41 of 64 (64%) passively reported cases were eligible for follow-up by local health workers.	Health center resource (budget, transport, personnel, etc.) planning for focus investigation should utilize past year’s case estimates, adjusted downwards by 20–30% to account for ineligible cases.
Some cases diagnosed at a health center came from outside the health center catchment area.	Focus investigation team deployment may be implemented locally from the health center but management and oversight need to occur at a district level or higher. District and provincial level personnel should be equipped to work across catchments areas and districts to deploy health center (or central level) teams to track cases that were diagnosed elsewhere.
Some cases could not be found during follow-up.	Include additional information in the notification reporting that would facilitate locating index cases, such as village name, phone number, or name of guardian in the case of a child.
Fidelity (adherence) was lower than in other 1-3-7 deployments. Case notification was only 61%.	Adherence monitoring and follow-up should happen at a central (district or provincial) level and reporting structures should be built such that central-level personnel are incentivized to follow-up with local teams conducting 1-3-7 focus investigation.
Reporting duration was long (48 minutes on average) and longer in areas with poor network connectivity. Network connectivity differed by area and provider.	Central-level staff should be aware of network connectivity in areas implementing 1-3-7 to understand when and why to expect delays in reporting. SMS aggregator platforms (TextIt) should be compatible with all network types so that reporters may use their network of choice.
Some cases diagnosed in the health center on the weekend were missed.	Staff must be designated in a health center to send and review notifications in real time, including weekends.
RCD completion, household coverage, and average households visited per follow-up were all substantially higher in zones implementing 1-3-7 compared to the control zones. 26% of eligible households in interventions zones were visited whereas only 8% of households in control zones were visited.	Real-time, case-specific, digital reporting and surveillance is necessary to reach more households.
Household distance from health centers emerged as a barrier to feasibility (CHWs could not get there as easily) but also the equity of the 1-3-7 program (more of these households were missed).	CHWs need incentives or support in travelling farther distances. When onboarded, it should be considered if the CHW owns a bicycle .
CHW belief that there was active transmission in their area was associated with shorter reporting times. CHWs were committed to their work and the goal of malaria elimination, and some requested receiving more information on malaria in their area.	Provide CHWs training on, and access to, malaria data platforms, such as the 1-3-7 R-Shiny dashboard that are relevant to their catchment area.
Talk time allowances enabled reporting and incentivized CHWs.	Provide health workers requisite allowance or other incentive for reporting via SMS.
As with other community-based interventions, men and age groups beyond childhood are most likely to be missed.	Distinct but complementary surveillance strategies should be introduced to include hard-to-reach groups.

Maximizing penetration (scale-up) and adoption in other low malaria transmission settings will be aided by the high acceptability of 1-3-7 focus investigation, but there are important human resource and financial costs that programs must plan for prior to implementing 1-3-7 focus investigation. First, managing the R-Shiny dashboard and SMS reporting, the associated databases, and their integration requires ongoing technical expertise. Second, to maintain timely notifications, staff must be available to send and review notifications in real time, including weekends. Lastly, budgets must support allocated mobile phone credit, CHW participation, and general programmatic costs to drive fidelity and adoption. One limitation of this work is the exclusion of cost from the selected implementation outcomes. An understanding of cost is critical to contextualizing recommendations and budget allocation is a key determinant of programmatic success. The authors conducted an informal cost analysis for programmatic dissemination but not a formal costing study. Another limitation is that acceptability was measured through the perspectives of health care staff and volunteers but did not include the perspectives of those served by the healthcare system. However, studies on community perceptions of malaria interventions in southern Africa show they are broadly welcomed [37, 38].

Perhaps the most significant limitation of the 1-3-7 focus investigation strategy in Zambia was the RCD intervention itself. Although a WHO-recommended intervention in low-transmission settings, the detection of few RDT positive individuals indicates the intervention was inefficient in this very low transmission setting [15]. Elsewhere, as 1-3-7 focus investigation is deployed, relative transmission intensity should be considered when selecting the day 7 intervention. In southern Zambia and similar areas, day 7 focus investigation and response could include interventions that target subpatent infections in both index and neighboring households, such as reactive focal vector control or focal mass drug administration, which were effective in a cluster-randomized trial in a low transmission endemic setting in Namibia [36]. Focal community sensitization could also be conducted, especially in communities where there is access to but low-use of malaria control interventions. As day 7 interventions are added or changed, their fidelity and coverage should be monitored, as these require adept commodity management and resource mobilization. WHO and country policies currently recommend interventions based on incidence and prevalence thresholds and future research could explore further sub-dividing low transmission areas to explore what interventions are successful at the extreme ends of low malaria transmission.

## Conclusions

This study may be the first to apply an implementation framework to 1-3-7 focus investigation and explicitly evaluate both implementation and service outcomes as defined by Proctor et. al [29]. It provides critical proof of concept for an implementation strategy that digitizes 1-3-7 focus investigation while uncovering aspects of the design that should be adapted and monitored in different implementation contexts. In addition to these findings, the study used a testable implementation approach through measurement of feasibility, fidelity, acceptability, efficiency, and equity outcomes, which are appropriate and important measures in early-to-mid-implementation. This framework should be expanded to include additional outcomes, such as cost, to assess implementation as 1-3-7 focus investigation is deployed elsewhere in Zambia and southern Africa. Scale-up and deployment should be based upon the major findings of this work: 1) technical system design should consider budget and infrastructural constraints; 2) adherence-focused management strategies should be deployed and sensitive to feasibility; 3) response interventions should be spatially focused, sensitive to local transmission levels, and explore alternatives to RCD; and 4) complementary surveillance should target hard-to-reach populations.

## Supporting information

S1 Checklist This document contains affirmations at to PLOS’ policy on inclusivity in global research aims. (DOCX)

## List of abbreviations

CHW: community health worker
CI: confidence interval
dbM: decibels per milliwatt
DO: district officer
EHT: environmental health technician
GMS: Greater Mekong subregion
ICEMR: International Centers of Excellence for Malaria Research
IQR: interquartile range
OR: odds ratio
OPD: Outpatient department
RAVC: reactive focal vector control
RCD: reactive case detection
rfMDA: reactive focal mass drug administration
SMS: short-messaging-service
SRQR: Standards for Reporting Qualitative Research
StaRI: Standards for Reporting Implementation Studies
WHO: World Health Organization
